# Trends in parallel application to emergency medicine residency between 2009 and 2023

**DOI:** 10.1002/aet2.70043

**Published:** 2025-05-08

**Authors:** Udoka Oji, Samuel Bunting, Nitin Vidyasagar, Emily Jameyfield, Paul Kukulski

**Affiliations:** ^1^ Section of Emergency Medicine, Department of Medicine University of Chicago Medicine Chicago Illinois USA; ^2^ Department of Psychiatry and Behavioral Science University of Chicago Medicine Chicago Illinois USA; ^3^ Pritzker School of Medicine University of Chicago Chicago Illinois USA; ^4^ Department of Emergency Medicine Yale School of Medicine New Haven Connecticut USA

## Abstract

**Objectives:**

The residency match can be a stressful and costly period for applicants. A notable portion of applicants choose to apply to multiple specialties during the residency match process, known as “parallel applying.” This study investigated the trends in parallel applications among emergency medicine (EM) residency applicants from 2009 to 2023.

**Methods:**

This is a retrospective analysis on data obtained from the Association of American Medical Colleges (AAMC) Electronic Residency Application System (ERAS). The data set comprised applicants who applied either exclusively to EM or to EM in combination with other specialties. Variables analyzed included the number of applications submitted and the frequency of parallel applications across different applicant groups including international medical graduates (IMGs), U.S. international medical graduates (USIMGs), and graduates from U.S. allopathic and osteopathic institutions.

**Results:**

The study included 64,095 applicant records corresponding to 57,572 unique individuals. The percentage of applicants to EM residency parallel applying decreased between 2009 (62.0%) and 2020 (43.0%) but subsequently increased through 2023 (62.6%). The total number of applications submitted by both single‐specialty and parallel applicants increased over the same period. The mean number of applications to EM programs by parallel applicants rose significantly, reflecting a broader trend of increasing application volumes across all applicant groups while the proportion of applications that were to EM programs decreased, especially following 2021.

**Conclusions:**

The trend of parallel applying among EM residency applicants slowly decreased from 2009 to 2021 and then increased through 2023, with the trend largely driven by an increase in the proportion of IMG applicants who were parallel applying. Understanding the motivations and impacts of parallel applications is crucial for developing strategies to support applicants and improve the matching process. Further research is needed to explore the factors influencing these trends and to inform policy and advising practices in medical education.

## INTRODUCTION

Deciding which specialty to apply to is the most significant career‐related decision a medical student makes. Further, applying to and matching into a residency program is one of the most stressful and costly periods of medical school.^1^, ^2^ To improve their chances of matching, some applicants apply to more than one specialty during an application cycle, referred to as “parallel applying.”^3^ There is limited literature describing parallel application within emergency medicine (EM). One previous study hypothesized varied motivations for parallel application among EM applicants, including difficulty choosing one specialty due to interest in diverse clinical experiences, prioritization of specific geographic location over specialty preference, and concerns about competition among EM applicants.^4^ However, no study to date has described how many applicants parallel apply to EM or trends in parallel application to EM over time.

Additionally, while applications to EM residency had been steadily increasing year after year,^5^ there was a sudden decrease in applications to EM during the 2023 Match cycle, resulting in 554 unfilled EM residency positions in 2023, up from 219 in the year prior.^6^, ^7^ Understanding trends in parallel application to EM residency is therefore vitally important not only to improve student advising, decrease applications, and alleviate applicant stress during the application season but also to further understand the current factors impacting the EM residency match.

Furthermore, in October 2024 the Council of Residency Directors in Emergency Medicine (CORD‐EM) announced that EM would transition to a proprietary application platform for the 2025/2026 application cycle.^8^ This follows recent decisions of other specialties like OB/GYN and plastic surgery to transition away from the Electronic Residency Application System (ERAS) to proprietary platforms. A majority of specialties are continuing to use ERAS for residency applications. Thus, if an applicant were to apply to more than one specialty (i.e., parallel apply), they would need to complete applications across multiple platforms, including payment of multiple application platform registration fees. While there are benefits of proprietary application systems, it is essential to understand the context in which they are introduced to best support applicants to EM residency as they develop their application strategy. The purpose of this study was to explore the prevalence of parallel applying among applicants to EM residency from 2009 to 2023 and to determine both the number of individual specialties included when applicants parallel apply to EM and the number of applications submitted when parallel applying.

## METHODS

### Study design

This study is a secondary analysis of data captured by the Association of American Medical Colleges (AAMC) through the ERAS, made available (for purchase) through a customized data report. This secondary analysis of anonymous administrative data was reviewed and determined to be nonhuman subjects research and was exempted from review by the institutional review board of the University of Chicago.

### Study setting and population

Data from the 2009/2010 through 2023/2024 application cycles (years 2009–2023) were provided by the AAMC through a custom ERAS data report. The ERAS data set reported data at the per‐applicant, per‐year, per‐specialty level. We restricted the data set to only those applicants who applied to residencies in EM, either alone (single‐specialty application) or in combination with one or more other, categorical specialties (parallel application).

### Study protocol

Within this restricted data set, the total number of residency applications submitted to each specialty to which an applicant applied in each year was calculated. We excluded applications to combined specialty residency programs (e.g., EM/anesthesiology). If an applicant was applying to a categorical specialty that also required a preliminary year in internal medicine (e.g., neurology), applications to preliminary positions in internal medicine were not counted as a parallel specialty. However, applications to *categorical* internal medicine programs were counted as additional specialties. Transitional year programs were counted as separate applications.

### Data analysis

Descriptive statistics were calculated to describe variables. Analyses were conducted for the overall group of applicants in the dataset as well as within the applicant subsets which were based on an applicants' type of medical training: public U.S. allopathic (MD) institutions, private U.S. allopathic institutions, osteopathic (DO) institutions, international medical graduate (IMG), or U.S. citizen IMG (USIMG). First, we calculated the percentage of applicants who were applying to EM in combination with at least one other specialty (parallel applying) in each year between 2009 and 2023. Next, we calculated the mean number of individual specialties to which applicants applied to when parallel applying with EM, separated by applicant group. The mean number of applications submitted to EM residencies among applicants who were parallel applying was calculated as was the proportion of the total number of applications that were to EM residency programs among the group of applicants who were parallel‐applying. All data management and analyses were completed with StataMP V17 (StataCorp).

## RESULTS

The ERAS data set contained 64,095 applicant records for applicants applying to EM residency between 2009 and 2023, which corresponded to 57,572 unique applicants (Table 1). Applicants were allowed to appear in more than 1 year of data to report the real‐world application environment in each year. The number of applicants to EM residency increased from 3,633 in 2009 to 5,893 in 2023. During this time period, the composition of the applicant pool shifted. The percentage of applicants to EM from public allopathic (28.9% [*n* = 1,049] vs. 20.2% [*n* = 1,192]) and private allopathic (18.0% [*n* = 654] vs. 12.9% [*n* = 758]) schools decreased. The percentage of IMG applicants to EM increased from 11.2% (*n* = 408) of applicants in 2009 to 20.2% (*n* = 1,191) in 2023 and the percentage of USIMG applicants increased from 23.9% (*n* = 869) to 25.2% (*n* = 1,485). Simultaneously, the proportion of osteopathic applicants to EM residency increased from 18.0% (*n* = 653) in 2009 to 21.5% (*n* = 1,267) in 2023.

**TABLE 1:** Number of applicants to EM residency by applicant group from 2009 to 2023.

	IMG	Osteopathic	US MD private	US MD public	USIMG	Total
2009	408 (11.2)	653 (18.0)	654 (18.0)	1,049 (28.9)	869 (23.9)	3,633
2010	423 (10.9)	733 (18.9)	719 (18.5)	1,071 (27.6)	941 (24.2)	3,887
2011	446 (10.8)	811 (19.6)	738 (17.9)	1,142 (27.6)	998 (24.1)	4,135
2012	258 (7.23)	813 (22.8)	684 (19.2)	1,119 (31.4)	693 (19.4)	3,567
2013	363 (8.73)	909 (21.9)	804 (19.3)	1,257 (30.2)	826 (19.9)	4,159
2014	416 (9.48)	892 (20.3)	816 (18.6)	1,300 (29.6)	966 (22.0)	4,390
2015	356 (8.42)	968 (22.9)	773 (18.3)	1,305 (30.9)	827 (19.6)	4,229
2016	220 (5.84)	951 (25.3)	755 (20.1)	1,233 (32.7)	607 (16.1)	3766
2017	197 (5.09)	1,023 (26.4)	801 (20.7)	1,303 (33.6)	550 (14.2)	3,874
2018	257 (5.91)	1,146 (26.4)	837 (19.3)	1,405 (32.3)	701 (16.1)	4346
2019	181 (4.42)	1,098 (26.8)	822 (20.1)	1,407 (34.3)	591 (14.4)	4,099
2020	217 (5.03)	1,106 (25.7)	870 (20.2)	1,462 (33.9)	656 (15.2)	4,311
2021	334 (6.70)	1,296 (26.0)	898 (18.0)	1,671 (33.5)	785 (15.8)	4,984
2022	411 (8.52)	1,225 (25.4)	817 (16.9)	1,459 (30.3)	910 (18.9)	4,822
2023	1,191 (20.2)	1,267 (21.5)	758 (12.9)	1,192 (20.2)	1,485 (25.2)	5,893

*Note*: Data are reported as *n* (%).

### Frequency of parallel application

**FIGURE 1:**
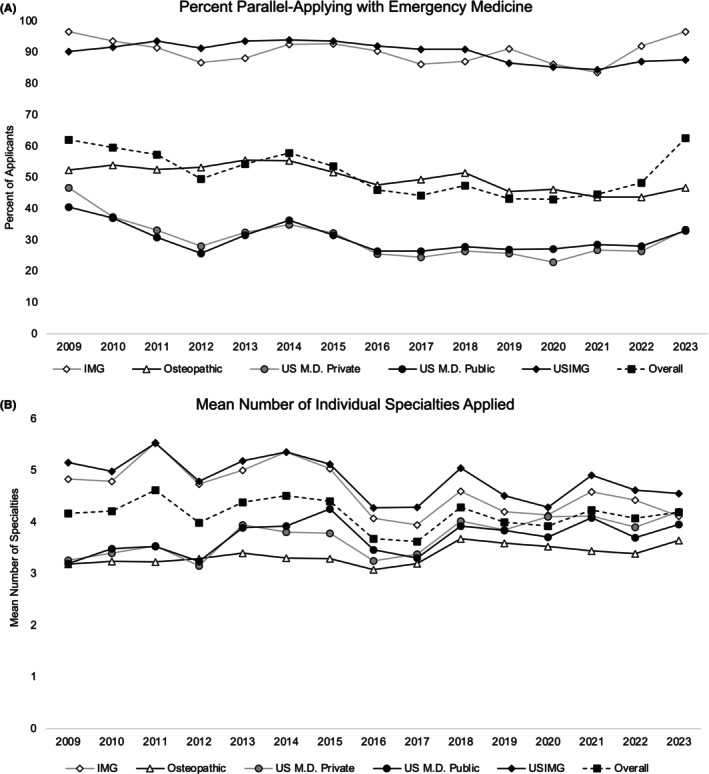
(A) Percentage of applicants who were parallel‐applying with EM between 2009 and 2023, separated by applicant group. (B) The mean number of individual specialties to which applicants parallel applying to EM applied between 2009 and 2023, separated by applicant group.

The frequency of parallel application to EM decreased between 2009 and 2022 for all applicant groups, with increases between 2022 and 2023 (Figure 1A). Overall, 62.0% of applicants to EM in 2009 were parallel applying, which remained largely stable at 62.6% in 2023; however, this represented an increase from 48.3% parallel applying in 2022 (Figure 1A). In all years, international applicants (IMGs and USIMGs) parallel applied to EM at the greatest frequency at 96.6% in both 2009 and 2023 for IMGs and 90.2%–87.6% between 2009 and 2023 for USIMG applicants. Applicants from U.S. private allopathic schools experienced the greatest percentage decrease in the frequency of parallel application (−13.3%), decreasing from 46.6% in 2009 to 33.4% in 2023. Applicants from U.S. public allopathic institutions also experienced decreases in the frequency parallel applying (40.6% vs. 32.9%) between 2009 and 2023. The frequency of osteopathic applicants parallel applying to EM decreased from 52.4% in 2009 to 46.7% in 2023.

### Specialty count

Overall, the mean number of individual specialties applicants applied to when parallel applying with EM remained relatively consistent between 2009 and 2023 (Figure 1B). Across all applicant groups, those who were parallel applying to EM applied to a mean of 4.17 (95% confidence interval [CI] 4.07–4.26) specialties in 2009, which increased minimally to 4.19 (95% CI 4.12–4.25) in 2023. IMG applicants experienced the greatest decrease in the mean number of specialties, from 4.83 (95% CI 4.61–5.05) in 2009 to 4.12 (95% CI 4.02–4.22) in 2023, a decrease of 0.71. USIMG applicants also experienced decreases in the mean number of specialties, from 5.15 (95% CI 4.98–5.32) in 2009 to 4.56 (95% CI 4.44–4.67) in 2023, a decrease of 0.59. Applicants from U.S. private allopathic (mean 3.26 [95% CI 3.07–3.45] vs. mean 4.21 [95% CI 3.91–4.50]), public allopathic (mean 3.19 [95% CI 3.04–3.35] vs. mean 3.95 [95% CI 3.74–4.16]), and osteopathic (mean 3.18 [95% CI 3.01–3.35] vs. mean 3.64 [95% CI 3.49–3.80]) institutions experienced increases in the mean number of specialties when parallel applying with EM.

### Application count

**FIGURE 2:**
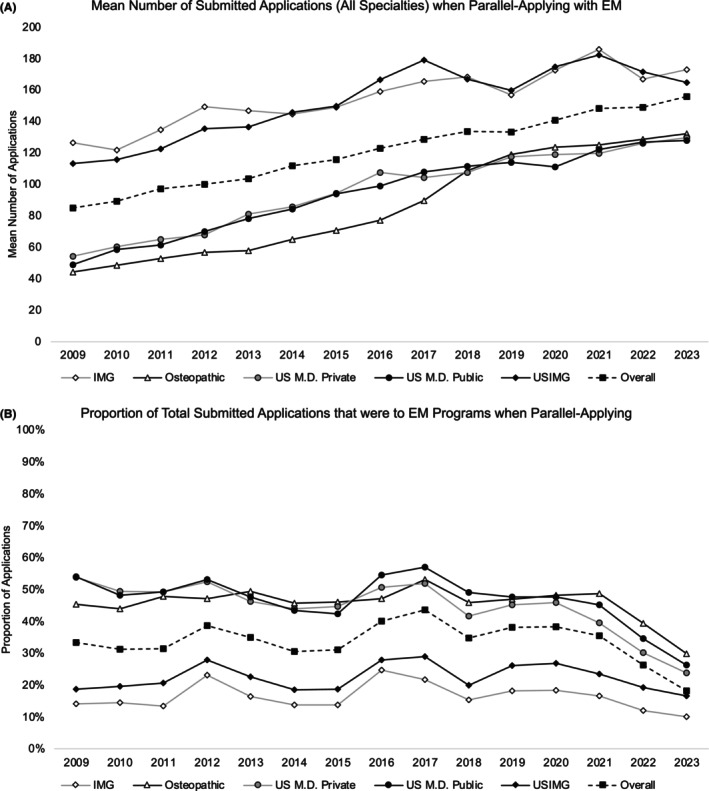
(A) Mean number of total applications (encompassing all specialties) submitted by applicants who were parallel applying with EM between 2009 and 2023, separated by applicant group. (B) The mean percentage of the total application count that were applications to EM programs among the groups of applicants who were parallel applying with EM.

The mean number of applications submitted to *all* specialties (EM and any others) among applicants parallel applying with EM increased for all groups between 2009 and 2023 (Figure 2A). Overall, the mean number of applications submitted increased from 84.9 (95% CI 81.9–88.1) in 2009 to 155.9 (95% CI 152.8–159.0) in 2023. A similar trend of increase was identified for IMG (mean 126.7 [95% CI 118.1–135.3] vs. mean 173.0 [95% CI 167.8–178.2]), USIMG (mean 113.2 [95% CI 107.1–119.4] vs. mean 165.0 [95% CI 158.8–171.1]) applicants parallel applying with EM, and these groups submitted the greatest mean number of applications in each year. U.S. applicants from private allopathic (mean 54.3 [95% CI 49.6–58.9] vs. mean 129.8 [95% CI 119.2–140.4]) and public allopathic (mean 48.9 [95% CI 45.7–52.0] vs. mean 128.0 [95% CI 121.4–134.7]) schools as well as osteopathic (mean 44.2 [95% CI 40.5–48.0] vs. mean 132.3 [95% CI 126.3–138.2]) schools also had increases in the mean number of total applications submitted when parallel applying with EM between 2009 and 2023.

The proportion of applications to EM programs also decreased between 2009 and 2023 within the group of applicants who were parallel applying with EM (Figure 2B). Across all applicant groups, the proportion of the total applications to EM programs decreased from 33.4% in 2009 to 18.3% in 2023. A similar pattern of decrease was observed across all applicant groups with the greatest decrease noted for applicants from public (54.0% vs. 23.8%, −30.1%) and private (54.0% vs. 26.4%, −27.5%) U.S. allopathic institutions, followed by applicants from osteopathic (45.4% vs. 29.9%, −15.5%) institutions. Decreases for IMG (14.3% vs. 10.1%, −4.1%) and USIMG (18.7% vs. 16.7%, −2.0%) applicants were noted but smaller in magnitude relative to applicants from domestic institutions.

### Specialty combinations

The number and percentage of applicants parallel‐applying to EM and other specialties each year from 2009 to 2023 are included in Table 2. The most frequently included specialties with EM when parallel applying in 2009 were internal medicine (*n* = 1,536, 68.2%), family medicine (*n* = 1,138, 50.6%), general surgery (*n* = 779, 34.6%), anesthesiology (*n* = 456, 20.3%), and transitional year programs (*n* = 816, 36.3%). In 2023, four of these five specialties remained the most frequent specialties in combination with EM among parallel applicants. In parallel with EM, there were 2,817 (76.4%) applicants to internal medicine, 2,172 (58.9%) to family medicine, 1,196 (32.4%) to general surgery, 1,264 (34.3%) to transitional year programs, and 649 (17.6%) to pediatrics.

**TABLE 2:** Number and percentage of applicants parallel applying to EM and other specialties from 2009 to 2023.

	2009	2010	2011	2012	2013	2014	2015	2016	2017	2018	2019	2020	2021	2022	2023
Anesthesiology	456 (20.3)	575 (24.8)	549 (23.2)	358 (20.3)	582 (25.8)	602 (23.7)	548 (24.2)	377 (21.8)	347 (20.2)	493 (23.9)	425 (24.1)	304 (16.4)	430 (19.3)	362 (15.5)	482 (13.1)
Child neurology	1 (0.04)	—	—	24 (1.36)	136 (6.03)	167 (6.57)	131 (5.79)	39 (2.25)	29 (1.69)	65 (3.15)	46 (2.6)	33 (1.78)	113 (5.08)	96 (4.12)	160 (4.34)
Dermatology	63 (2.8)	93 (4.01)	100 (4.22)	58 (3.28)	113 (5.01)	116 (4.56)	61 (2.69)	47 (2.72)	43 (2.51)	118 (5.72)	115 (6.51)	88 (4.75)	107 (4.81)	149 (6.39)	125 (3.39)
Diagnostic radiology	254 (11.3)	206 (8.89)	288 (12.2)	187 (10.6)	349 (15.5)	386 (15.2)	379 (16.7)	166 (9.6)	116 (6.76)	167 (8.1)	148 (8.38)	177 (9.55)	226 (10.2)	219 (9.4)	187 (5.07)
Family medicine	1138 (50.6)	1231 (53.1)	1407 (59.4)	921 (52.1)	1265 (56.1)	1509 (59.4)	1344 (59.4)	912 (52.7)	915 (53.4)	1159 (56.2)	951 (53.8)	995 (53.7)	1188 (53.4)	1274 (54.7)	2172 (58.9)
General surgery	779 (34.6)	803 (34.6)	885 (37.4)	580 (32.8)	814 (36.1)	939 (36.9)	830 (36.7)	497 (28.7)	491 (28.6)	702 (34.0)	565 (32.0)	565 (30.5)	734 (33.0)	830 (35.6)	1196 (32.4)
Internal medicine	1536 (68.2)	1548 (66.8)	1716 (72.5)	1159 (65.6)	1527 (67.8)	1763 (69.4)	1595 (70.5)	1162 (67.2)	1108 (64.6)	1459 (70.7)	1181 (66.8)	1272 (68.7)	1586 (71.3)	1631 (70.0)	2817 (76.4)
Interventional radiology (integrated)	—	—	—	—	—	—	—	—	71 (4.14)	19 (0.92)	16 (0.91)	107 (5.77)	178 (8)	20 (0.86)	153 (4.15)
Neurodevelopmental disabilities	—	—	—	9 (0.51)	8 (0.35)	1 (0.04)	5 (0.22)	3 (0.17)	2 (0.12)	13 (0.63)	3 (0.17)	4 (0.22)	7 (0.31)	3 (0.13)	15 (0.41)
Neurology	181 (8.04)	180 (7.77)	301 (12.7)	129 (7.3)	247 (11.0)	310 (12.2)	217 (9.58)	102 (5.9)	102 (5.95)	226 (11.0)	164 (9.28)	127 (6.85)	231 (10.4)	246 (10.6)	444 (12.0)
Neurosurgery	31 (1.38)	73 (3.15)	100 (4.22)	37 (2.09)	59 (2.62)	38 (1.49)	105 (4.64)	28 (1.62)	9 (0.52)	22 (1.07)	17 (0.96)	26 (1.4)	24 (1.08)	37 (1.59)	132 (3.58)
Nuclear medicine	13 (0.58)	33 (1.42)	72 (3.04)	19 (1.07)	44 (1.95)	29 (1.14)	14 (0.62)	8 (0.46)	5 (0.29)	27 (1.31)	4 (0.23)	4 (0.22)	9 (0.4)	8 (0.34)	56 (1.52)
OB/GYN	247 (11.0)	227 (9.79)	270 (11.41)	167 (9.45)	230 (10.2)	296 (11.6)	190 (8.39)	112 (6.47)	88 (5.13)	181 (8.77)	121 (6.85)	123 (6.64)	144 (6.47)	217 (9.31)	245 (6.64)
Orthopedic surgery	126 (5.6)	194 (8.37)	171 (7.22)	112 (6.33)	106 (4.7)	230 (9.05)	133 (5.87)	85 (4.91	86 (5.01)	141 (6.83)	167 (9.45)	163 (8.8)	162 (7.28)	145 (6.22)	177 (4.8)
Osteopathic neuromuscular medicine	—	2 (0.09)	2 (0.08)	—	1 (0.04)	1 (0.04)	2 (0.09)	1 (0.06)	—	2 (0.1)	6 (0.34)	18 (0.97)	49 (2.2)	22 (0.94)	39 (1.06)
Otolaryngology	100 (4.44)	77 (3.32)	66 (2.79)	50 (2.83)	85 (3.77)	42 (1.65)	86 (3.8)	27 (1.56)	49 (2.86)	121 (5.87)	23 (1.3)	64 (3.45)	24 (1.08)	23 (0.99)	121 (3.28)
Pathology	256 (11.4)	199 (8.58)	336 (14.2)	163 (9.22)	237 (10.5)	391 (15.4)	216 (9.54)	83 (4.8)	93 (5.42)	222 (10.8)	95 (5.38)	91 (4.91)	172 (7.73)	203 (8.71)	237 (6.43)
Pediatrics	444 (19.7)	425 (18.3)	549 (23.2)	306 (17.3)	466 (20.7)	538 (21.2)	447 (19.7)	230 (13.3)	236 (13.8)	387 (18.8)	231 (13.1)	245 (13.2)	373 (16.8)	365 (15.7)	649 (17.6)
Physical medicine & rehabilitation	218 (9.68)	255 (11)	324 (13.7)	99 (5.6)	96 (4.26)	109 (4.29)	130 (5.74)	97 (5.61)	66 (3.85)	80 (3.88)	96 (5.43)	74 (3.99)	152 (6.83)	160 (6.86)	170 (4.61)
Plastic surgery^a^	26 (1.16)	23 (0.99)	21 (0.89)	54 (3.05)	51 (2.26)	11 (0.43)	110 (4.86)	21 (1.21)	28 (1.63)	80 (3.88)	14 (0.79)	13 (0.7)	25 (1.12)	22 (0.94)	34 (0.92)
Preventive medicine	—	48 (2.07)	65 (2.75)	25 (1.41)	22 (0.98)	25 (0.98)	20 (0.88)	17 (0.98)	14 (0.82)	18 (0.87)	20 (1.13)	11 (0.59)	31 (1.39)	28 (1.2)	33 (0.89)
Psychiatry	315 (14.0)	320 (13.8)	449 (19.0)	227 (12.8)	346 (15.4)	487 (19.2)	346 (15.3)	167 (9.65)	149 (8.69)	316 (15.3)	209 (11.8)	185 (9.98)	253 (11.4)	301 (12.9)	484 (13.1)
Radiation oncology	90 (4)	45 (1.94)	55 (2.32)	12 (0.68)	55 (2.44)	49 (1.93)	36 (1.59)	8 (0.46)	27 (1.57)	48 (2.33)	92 (5.21)	91 (4.91)	116 (5.21)	110 (4.72)	191 (5.18)
Thoracic surgery (Integrated)	4 (0.18)	15 (0.65)	13 (0.55)	13 (0.74)	13 (0.58)	20 (0.79)	14 (0.62)	10 (0.58)	33 (1.92)	10 (0.48)	11 (0.62)	1 (0.05)	61 (2.74)	12 (0.51)	14 (0.38)
Urology	33 (1.47)	31 (1.34)	21 (0.89)	24 (1.36)	26 (1.15)	31 (1.22)	31 (1.37)	12 (0.69)	14 (0.82)	11 (0.53)	18 (1.02)	21 (1.13)	30 (1.35)	16 (0.69)	26 (0.7)
Vascular surgery (integrated)	19 (0.84)	82 (3.54)	98 (4.14)	13 (0.74)	66 (2.93)	121 (4.76)	74 (3.27)	9 (0.52)	19 (1.11)	87 (4.22)	101 (5.72)	80 (4.32)	25 (1.12)	22 (0.94)	130 (3.52)
Transitional year	816 (36.3)	776 (33.5)	728 (30.8)	577 (32.6)	730 (32.4)	774 (30.5)	686 (30.3)	478 (27.6)	417 (24.3)	641 (31.1)	473 (26.8)	528 (28.5)	743 (33.4)	653 (28.0)	1264 (34.3)

*Note*: Number of applicants to EM residency who were parallel applying and also applying to residency programs in the specialty listed in the far left column.

^a^ Plastic surgery contains both categorical and integrated programs.

## DISCUSSION

This study reveals an interesting trend: the prevalence of parallel applying declined until 2022 where there was a sharp increase, primarily due to a surge in IMG parallel applicants. Simultaneously, the total percentage of U.S. MD applicants to EM decreased. These trends collectively suggest that while the total number of EM applications rebounded after 2021, this recovery was largely driven by IMG applicants. Further, while the majority of the applicants who are parallel applying to EM are IMG and USIMG applicants, a large percentage of U.S. allopathic and osteopathic applicants continue to parallel apply. Additionally, the proportion of applications to EM programs among those who were parallel applying declined precipitously between 2021 and 2023 possibly implying that EM has become less popular and may be serving as applicants “backup” specialty.

Given the overall lower match rate to U.S. residencies (all specialties) for IMG and USIMG compared to U.S. applicants,^9^ it is reasonable to assume that the higher prevalence of parallel applying to residency in this cohort is largely driven by a desire to avoid going unmatched in any specialty, not just going unmatched in EM during the process. This hypothesis could explain the recent increase in parallel applications to EM, bringing it back to 2009 levels, as the number of IMG applicants to EM in general has greatly increased, and the trend would suggest that the increase in IMG applications, specifically those who are parallel applying, is driven not by IMG applicants specifically interested in EM but those wishing to avoid going unmatched in the United States in any specialty. This is further supported by the finding that USIMG and IMG applicants had the smallest percentage decreases in the proportion of applications to EM programs among those who were parallel applying. However, much is still unknown and further study is required to determine the reason for such a high prevalence of parallel application among IMG applicants.

Despite the overall trend being driven by IMG applicants, as of 2023 between 33% and 47% of U.S. MD and U.S. DO applicants still parallel apply to EM. It is unclear whether the primary driving force for this phenomenon is difficulty in choosing a specialty, fear of going unmatched for residency, or both. Further research is needed to determine the reasons behind the decision to apply to multiple specialties so that EM education leaders can determine how best to work with applicants at their institutions to help avoid parallel application. The decrease in parallel application among U.S. applicants over the 15‐year study period is encouraging, and further understanding the factors driving the trend will help advisors when working with residency applicants prior to application.

While there will always be some applicants who choose to parallel apply, there are multiple reasons to aim to decrease the overall number. First, applying to residency remains very costly for applicants.^1^ While the AAM Cupdated the fee structure for ERAS applications in 2024 to make it more affordable (including a fee assistance program), the cost per application still increases with the number of applications that are submitted, meaning parallel applicants bear an even greater financial load due to the increased number of applications to multiple specialties as fees are assessed per specialty.^10^ This financial strain extends beyond application fees when one takes into consideration other expenses such as standardized examinations, professional attire, transcript release, and Match registration.

Second, EM residencies have an interest in recruiting residents who truly want to become EM physicians. There are negative consequences for both the resident and the program when a resident chooses to change specialties midresidency or leave medicine altogether due to poor specialty fit. Additionally, it is well‐known that EM residents have a high rate of burnout,^11^, ^12^ and a resident who is unhappy with their specialty choice is likely to experience this at higher rates.^12^

Third, the transition to a proprietary application system for the 2025–2026 EM residency match coincides with an increase in the overall proportion of applicants to EM who are parallel applying. For these applicants, they will need to upload their application materials to two platforms and may face additional expense depending on the pricing structure and number of applications submitted. Further study will be need to see how this will affect the frequency of parallel application with EM; however, results from this study demonstrate that it may be an issue for a large number of applicants, especially international applicants.

Finally, following the 2023 Match, there were 554 unfilled residency positions in EM and following the 2022 Match there were 219 unfilled positions, when in prior years there had only been anywhere between 13 and 30 unfilled positions.^6^ This number decreased to 135 unfilled positions after the 2024 Match, following the large increase in IMG applicants.^13^ A joint statement by a broad body of EM organizations was released following the 2023 Match announcing the initiation of a Match task force to identify factors contributing to this new phenomenon of unfilled residency spots and to offer solutions to address these factors.^14^ While the trend appears to have reversed (yet not reverted to pre‐2022 levels), both the increase in total applications and the decrease in unmatched positions in 2024 seem to be driven by the increased number of IMG applicants. Additionally, parallel applications may reflect a significant contributor to the disparity between applications and Match results if applicants are choosing specialties they ultimately deem more favorable over EM. However, further studies taking a qualitative approach regarding parallel applications are required to delve deeper into the motivations behind parallel applying and applicant selection of specialties.

## LIMITATIONS

One of the key strengths of this study is its use of a large sample of applicants, which allows for a comprehensive, population‐level examination of the trends in parallel applications over time. However, the data used in the analysis did not include other variables that may have also contributed to parallel application including standardized examination scores, away rotations, or other elements used in the resident selection process. Additionally, this study does not account for the individual motivations and circumstances that may be driving applicants to submit parallel applications.

## CONCLUSIONS

The number of applicants who parallel apply to multiple specialties with emergency medicine slowly decreased from 2009 to 2021 and then increased back to 2009 levels by 2023. The data in this study demonstrates that, while a large percentage of U.S. allopathic and osteopathic applicants continue to parallel apply, this trend was driven by a notable increase in international medical graduate student applications. Further studies are needed to determine the motivations behind parallel applying and to explore factors that influence the Match process for different applicant demographics to aid educators and program directors with developing more effective and equitable strategies for matching applicants with residency programs.

## FUNDING INFORMATION

This project was supported by the Department of Psychiatry and Behavioral Neuroscience and the Office of Diversity of the Biological Sciences Division of the University of Chicago. No other funding was received to support this project.

## CONFLICT OF INTEREST STATEMENT

This material is based upon data provided by the Association of American Medical Colleges (‘AAMC’). The views expressed herein are those of the authors and do not necessarily reflect the position or policy of the AAMC. Dr. Bunting reports receiving unrestricted research funding from Gilead Sciences for work unrelated to the present study. The remaining authors declare no conflicts of interest.

## Data Availability

Data cannot be made available due to limitations of a signed data use agreement with the AAMC.
